# A Review and Update on Waterborne Viral Diseases Associated with Swimming Pools

**DOI:** 10.3390/ijerph16020166

**Published:** 2019-01-09

**Authors:** Lucia Bonadonna, Giuseppina La Rosa

**Affiliations:** Italian National Institute of Health, Viale Regina Elena, 299-00161 Rome, Italy

**Keywords:** adenovirus, enterovirus, hepatitis A virus, norovirus, swimming pool, waterborne disease

## Abstract

Infectious agents, including bacteria, viruses, protozoa, and molds, may threaten the health of swimming pool bathers. Viruses are a major cause of recreationally-associated waterborne diseases linked to pools, lakes, ponds, thermal pools/spas, rivers, and hot springs. They can make their way into waters through the accidental release of fecal matter, body fluids (saliva, mucus), or skin flakes by symptomatic or asymptomatic carriers. We present an updated overview of epidemiological data on viral outbreaks, a project motivated, among other things, by the availability of improved viral detection methodologies. Special attention is paid to outbreak investigations (source of the outbreak, pathways of transmission, chlorination/disinfection). Epidemiological studies on incidents of viral contamination of swimming pools under non-epidemic conditions are also reviewed.

## 1. Introduction

Swimming pools have been implicated in the transmission of infections. The risk of infection has mainly been linked to fecal contamination of the water, generally due to feces released by bathers or to contaminated source water. Failure in disinfection has been recorded as the main cause of many of the outbreaks associated with swimming pools.

The majority of reported swimming pool-related outbreaks have been caused by enteric viruses [1,2]. Sinclair and collaborators reported that 48% of viral outbreaks occur in swimming pools, 40% in lakes or ponds, and the remaining 12% in fountains, hot springs, and rivers (4% each) [1].

Viruses cannot replicate outside their host’s tissues and cannot multiply in the environment. Therefore, the presence of viruses in a swimming pool is the result of direct contamination by bathers, who may shed viruses through unintentional fecal release, or through the release of body fluids such as saliva, mucus, or vomitus [3]. Evidence suggests that skin may also be a potential source of pathogenic viruses.

## 2. Materials and Methods

We carried out a comprehensive literature review aimed at investigating waterborne viral outbreaks linked to swimming pools, to explore the etiological agents implicated, pathways of transmission, associations between indicator organisms and disease, and key issues related to chlorination/disinfection procedures. Viral outbreaks are summarized in Table 1. The presence of enteric viruses in swimming pools under non-epidemic conditions was also reviewed. Different databases (Scopus, PubMed, and Google Scholar) were accessed using the terms norovirus, Norwalk virus, adenovirus, enterovirus, echovirus, coxsackievirus, and hepatitis A, in combination with terms recreation, swimming, pool, and water.

## 3. Viral Outbreaks Related to Swimming Pools

### 3.1. Adenovirus Outbreaks (N° = 15)

Adenoviruses are the enteric viruses most commonly associated with swimming pool-related outbreaks. Human adenoviruses (HAdVs) belong to the Adenoviridae family and are classified into seven species (A to G) and more than 90 types [4]. HAdVs are of major public health importance and can result in a variety of clinical manifestations, including gastroenteritis, respiratory, ocular and urinary tract infections [5]. Illnesses are common and ubiquitous with a worldwide distribution. HAdVs are highly stable in the environment and can survive for prolonged periods in water [6]. Transmission in swimming pools can occur by ingestion, direct contact with contaminated water, or through the inhalation of aerosol [7].

A Brazilian study recently detected HAdVs in *Acanthamoeba* isolated from water samples collected from swimming pools [8]. HAdVs were found in 62.5% (10/16) of amoebae with DNA copies up to 5.1 × 10^5^ per milliliter, suggesting that *Acanthamoeba* may act as a reservoir and promote HAdV transmission through water.

The first swimming pool-related outbreak, published in 1953, described a 1951 outbreak in Greeley, Colorado, which thus came to be known as the “Greeley epidemic”. The outbreak, involving 206 cases, caused a combination of symptoms, such as acute conjunctivitis, pharyngitis, muscle pain, and fever [9]. Between 25% and 50% of children swimming in the pool were affected. The transmission apparently occurred either by contact with contaminated objects, such as toys, or while swimming in a pool. The water was heavily chlorinated, with residual chlorine being close to 0.4 parts per million. No definite pathogen was identified at the time of publication. Serum samples from Greeley patients were later tested and showed a specific neutralizing antibody response to HAdV type 3 [10].

In 1954, an epidemic of pharyngeal-conjunctival fever occurred in Washington, D.C., with symptoms similar to those of the Greeley epidemic [10]. Over 300 cases were documented, with acute respiratory illness characterized by one or more of the following symptoms: fever, pharyngitis, and conjunctivitis. Cases occurred in all age groups, but predominantly in children. Adenovirus type 3 was isolated in 80 of 300 patients from eye, throat washings and stools. The disease occurred at different sites: in a children’s summer day camp, in an orphanage, and in two residential neighborhoods. The suspected, but never confirmed, source of the epidemic was a swimming pool, even if cases due to direct contact were also recorded in houses and hospitals. The pool, chlorinated by hand, showed a low level of residual chlorine. Bell and collaborators were the first to suggest the term pharyngoconjunctival fever for this disease.

In August 1955, 112 cases of pharyngoconjunctival fever occurred in Toronto, linked to an indoor swimming pool. Seventy-four of the cases were children who had swum in the pool, while the others had swum in pools elsewhere, or had had direct contact with a case at home. Only one case had no history of either swimming or direct contact. Most of the children had pharyngitis, fever, malaise, and muscle pain. Conjunctivitis was absent or minimal in children, but was the main cause of discomfort in adults [11].

Another outbreak of pharyngoconjunctival fever was documented in August and September 1959 in Saitama Prefecture, Japan, among students of a primary and a middle school [12]. Epidemiological investigations suggested that the outbreak was mainly due to the contamination of a swimming pool used by the students of both schools. A total of 358 students were affected: 248 primary school students (attack rate, 20.6%) and 110 middle school students (attack rate, 19.2%). Laboratory findings suggested that the epidemic was due to HAdVs 3 and 7.

Foy and coworkers described an outbreak of pharyngoconjunctival fever (45 cases) in two swimming teams in Washington in 1966. Adenovirus type 3 was the etiological agent [13]. Most of the infected children had fever, pharyngitis, conjunctivitis, and diarrhea. In adults, symptoms were milder, with a high incidence of conjunctivitis. The attack rate was 65% and 67% for the two teams, respectively. Children had swum in the early morning, when the chlorinator of the pool was switched off, to avoid eye irritation with chlorine. Within one week, 25 of the 36 exposed swimmers became ill. Children swimming in the afternoon, when chlorination was still working, did not get sick. The infection was shown to spread in families having index cases (20 infected contacts). Analyses of water were done approximately 14 days after the presumed exposure. For this reason, attempts to isolate the virus from the water failed.

An outbreak of acute conjunctivitis due to HAdV type 7 occurred in Kansas, USA in 1973: 44 cases and one hospitalization were documented [14]. Eye symptoms predominated (red or pink eyes, swollen eyes), but a variety of other signs were also noted (mainly fever, headache, and nausea). A school swimming pool was identified as the source of infection. Chlorine concentration was low due to an equipment failure. The epidemic was easily controlled by raising the pool’s chlorine level. Unfortunately, tests for viruses and bacterial indicators were carried out after super-chlorination and consequently results were negative.

In 1977, two outbreaks associated with swimming pools occurred in Georgia, USA. The first, due to HAdV type 3, involved at least 105 cases [15], with patients showing different symptoms, including sore throat, fever, headache, anorexia and conjunctivitis. In this case, a private swimming pool was the source of infection. A temporary malfunction in the water filtration system of the pool associated with inadequate chlorine levels was recorded. Both waterborne and person-to-person transmission occurred. In the second outbreak, HAdV type 4 was recognized as the etiological agent of pharyngoconjunctival fever in 72 persons [16]. An insufficient amount of chlorine was found in the water of the pool. To stop the spread of infection, the pool was closed during the summer and adequately chlorinated. Adenovirus was recovered from the water sampled from the pool.

In Oklahoma, USA, an outbreak of pharyngitis caused by HAdV type 7a was recorded in 1982 among 77 children attending a swimming pool [17]. Symptoms included conjunctivitis, fever, sore throat, headache, and abdominal pain. Two cases were hospitalized with dehydration from persistent vomiting. A malfunction of the automatic pool chlorinator was identified as the cause of the outbreak. In fact, during the two weeks preceding the epidemic, its failure forced the pool operator to manually add chlorine to the pool.

Another outbreak was recorded in 1995, in Greece, where 80 athletes under 18 years of age presented with fever, conjunctivitis, sore throat, weakness, and abdominal pain, after swimming in a pool [18]. Seven athletes were hospitalized. Virological analyses on clinical samples were not performed and the illness was attributed to HAdV on the basis of clinical symptoms alone. Water samples from both the pool and the distribution system were tested for HAdV, enterovirus, and hepatitis A virus by molecular methods. The water of the pool tested positive for HAdV and negative for the other viruses, demonstrating its role as the source of infection. Samples from the water system were negative for all viruses tested. Chlorine levels were found to be low, probably due to a malfunctioning of the pool chlorination system.

Five HAdV outbreaks associated with swimming were recorded in the 2000s.

In the year 2000, an outbreak of pharyngoconjunctival fever occurred in North Queensland, Australia, where, after a school camp, 34 children aged 4–12, got sick [19]. In addition to primary cases acquired at the camp (N° = 25), nine other cases were acquired within the households. The school camp had a large saltwater swimming pool. Adenovirus 3 was isolated from eye and throat swabs. A PCR analysis of water samples for HAdV did not yield positive results. It was, however, demonstrated that the pool was not properly maintained, and that the level of residual chlorine was inadequate.

An outbreak of pharyngoconjunctival fever affecting 59 children under 15 was recorded in a municipality of Northern Spain in July 2008 [20]. Forty-three cases were recognized as primary cases, all of whom attended a municipal swimming pool. The remaining 15 children were secondary cases, which had been in close contact with a primary case. Adenovirus type 4 was detected in pharyngeal swabs. Electrical system failures causing the intermittent breakdown of the pool’s bromine dosing pumps and the slowing down of water circulation were assumed to have been the cause of the outbreak. Swimming was only allowed after the disinfection system was restored and appropriate concentrations of bromine were reached. Due to logistic problems, no water samples were taken from the swimming pool for virological analysis.

In 2011, children (4–9 years old) who had attended a swimming training center in Eastern China showed symptoms of pharyngoconjunctival fever. Adenovirus type 3 was recognized as the etiological agent [21]. A total of 134 cases were confirmed from among 900 amateur swimmers, with an incidence of 14.9%. Fourteen hospital admissions were documented. Fever, tonsillitis, sore throat, headache, sneezing, cough, conjunctivitis, fatigue, and diarrhea occurred among the bathers. The low level of residual chlorine in the water, along with excessive crowding in the pool were suggested as having caused the epidemic.

In the same year, in a primary school in Taiwan, an outbreak of HAdV infection occurred among 373 students, with four hospitalizations [22]. Most of the students attended a swimming course in two swimming facilities outside the school and presented with fever and symptoms of upper respiratory tract infection. Other symptoms included diarrhea, vomiting, skin eruptions and conjunctivitis. Throat swabs of affected students were tested for influenza virus, adenovirus, respiratory syncytial virus, coronavirus, metapneumovirus, parainfluenza types 1–4, and herpes simplex virus. Samples were found positive only for HAdV type 7. Water samples were not obtained from any of the facilities for virological analysis.

In 2013, an outbreak of pharyngoconjunctival fever involved 55 people (49 students and six staff) at a university in Beijing, China [23]. Fifty patients (91%) attending the same swimming pool two weeks before the onset of symptoms were considered primary cases. The other five subjects (9%) who had not swum in the pool were defined as secondary cases (person-to-person transmission). Human AdV type 4 was identified from both eye and throat swabs of the patients and from concentrated swimming pool water samples. Gene sequences obtained from the water samples exhibited a 100% match with the sequences obtained from swab samples. Control measures included the emptying and closing of the pool, and the disinfection with a high dose of sodium hypochlorite (500 mg/L).

### 3.2. Enterovirus Outbreaks (N° = 6)

Enterovirus is a genus in the family Picornaviridae, consisting of four human enterovirus species. Enteroviruses can cause many illnesses, including paralysis, meningitis, and cardiomyopathy, although most infections are asymptomatic or cause less severe conditions, such as colds and fever. A number of reports have described enterovirus infections linked to swimming pools.

The first enterovirus swimming pool-related outbreak occurred in 1987, at a municipal pool in Colorado, USA. Twenty-six children presented with fever along with at least one additional symptom such as malaise, headache, stomachache, nausea, or diarrhea [24]. It was found that the pool chlorination system was operating improperly, with chlorine levels close to zero. Stool specimens collected from the children affected were tested for common enteric bacterial pathogens (*Salmonella*, *Shigella*, *Aeromonas*, and *Campylobacter*), but not for viruses. Enterovirus was suggested as a likely etiological agent based on clinical manifestations, course of disease, incubation time, and the exclusion of likely bacterial pathogens.

An enterovirus outbreak occurred in Ireland in 1992, with 46 cases experiencing vomiting, diarrhea, and headache after attending an outdoor swimming pool in a small seaside village. One subject had vomited into the pool, and echovirus 30 was isolated from this case and from six other cases. Chlorine levels were found to comply with health standards, but were inadequate to contain the risk of infection from vomitus [25].

Another echovirus 30 outbreak occurred in Rome, Italy, in late 1997 [26]. Children from two schools showed clinical manifestations after swimming in a pool. Twenty children had meningitis-like symptoms (fever, headache, and vomiting), and six of them were hospitalized. Other 48 children had respiratory symptoms consistent with enterovirus infection. Echovirus 30 was isolated from the cerebrospinal fluid and stools of the hospitalized children. Based on the epidemiological characteristics, it was hypothesized that person-to-person transmission occurred both at the swimming pool and in a number of classrooms. Data on chlorination at the time of the outbreak were not available. Virological analysis of pool water was performed one month after the outbreak, but yielded no positive results.

In South Africa, an outbreak involving 90 children occurred following a summer camp in 2001 [27]. Camp activities included swimming and other aquatic sports. Symptoms included mainly headaches, sore eyes, and/or abdominal discomfort, with one case of vomiting. Four children were hospitalized for meningitis. Echovirus 3 was detected in cerebrospinal fluid and stool samples from symptomatic and asymptomatic children. The presence of viruses in the pool was not investigated. Water contamination was confirmed through a total coliform count.

In Germany, 215 cases of aseptic meningitis were recorded from July to October 2001 [28]. Swimming in a public, nature-like pond was identified as a risk factor for disease. Up to 1500 people visited the pond each day during the summer holidays. Echovirus 3 and 30 were detected in cerebrospinal fluid samples taken from some of the patients. An echovirus 30 sequence obtained from one water sample collected from the pond showed a high level of genetic similarity (99% nucleotide homology) with sequences obtained from patient isolates.

In August 2003, an outbreak of meningitis occurred among campers staying at a campground in Connecticut, USA [29]. A total of 12 cases of aseptic meningitis, four hospitalized patients and 24 cases of enterovirus-like illness with symptoms such as headache, neck stiffness, photophobia, sore throat, chills, or exanthema were identified. Echovirus serotype 9 was detected in cerebrospinal fluid samples from three of the patients. The spread of the virus was associated with swimming in a crowded pool, which had low chlorine levels. As a result, the pool water was intermittently contaminated with enterovirus.

### 3.3. Hepatitis a Virus Outbreaks (N° = 3)

Hepatitis A is a virus causing mild to severe liver disease. Globally, there are an estimated 1.4 million cases of hepatitis A every year. The virus is transmitted mainly via the fecal/oral route through the ingestion of contaminated food and water, or through direct contact with an infected subject. There is evidence to suggest that hepatitis A can be acquired by swimming in contaminated water.

In September 1979, an outbreak of hepatitis A affecting 56 children (5–17 years old) and causing 31 hospitalizations was recorded in Hungary [30]. All of the children swam in a pool at a summer camp. The pool was a non-chlorinated thermal pool/spa, which was overcrowded during the month of August. It was concluded that crowding and poor hygienic conditions, with a suspected accidental fecal release, contributed to the outbreak.

Another outbreak of hepatitis A was described in the USA during 1989. It involved 20 cases, probably associated with a public swimming pool [31]. It was hypothesized that a cross-connection between a sewage line and the pool water intake line may have been the cause of the outbreak. According to another hypothesis, it was a swimmer who contaminated the water in the pool. However, disinfectant levels in the pools met local standards.

Seven hepatitis A cases among children from six families were documented in Australia in 1997 [32]. The children had attended an outdoor spa pool treated with hydrogen peroxide solution. It was hypothesized that hepatitis A virus was shed by the index case in the spa pool, and subsequently ingested by the others, who became secondary cases. Virological analyses of water samples were not performed.

### 3.4. Norovirus Outbreaks (N° = 7)

Noroviruses (NoVs), formerly known as Norwalk-like viruses, are small viruses within the family *Caliciviridae*, subdivided into at least seven genogroups (GI–VII), with GI, GII, and GIV infecting humans. They are recognized as a major cause of sporadic and epidemic gastroenteritis in both industrialized and non-industrialized countries.

Outbreaks have been associated with a variety of settings including childcare centers, hospitals, nursing homes, cruise ships and restaurants. Noroviruses are mainly transmitted via the fecal-oral route through contaminated food or water. Norovirus-contaminated water—both recreational and drinking water—can thus lead to waterborne infections. A number of NoV swimming-pool related outbreaks have been described.

In 1977, an outbreak of acute gastroenteritis with the typical symptoms of vomiting, cramping, nausea and diarrhea was documented among 103 students and teachers at a primary school in Ohio, USA [33]. Serologic studies suggested infection by Norwalk virus to be the cause of the outbreak. The first cases recorded were caused by swimming in a contaminated pool, and a person-to-person transmission followed. The water of the pool tested negative for both bacterial and viral pathogens. Water contamination was linked to both the pool chlorinator, which was accidentally turned off at the time of the school visits, and a leak in the water supply pipes.

A large outbreak of gastroenteritis due to NoVs was recorded in July 2001 in Helsinki. It involved 242 people (children and adults) after bathing in an outdoor wading pool [34]. Norovirus and astrovirus were detected in both patient stool samples and pool water, with identical nucleotide sequences. The pool was found to be heavily contaminated with human fecal material carried from public toilets. The pool water had been manually chlorinated three times per week, thus not continuously. To control the outbreak, the pool was emptied, refilled, and the water was heavily chlorinated (up to 10 mg/L of free chlorine). For the subsequent swimming season, the pool was equipped with both a continuous chlorination system and a water filtration system.

In 2002, a NoV outbreak associated with a swimming pool was reported in Minnesota, USA. Thirty-six persons of three different youth sports teams became ill after swimming in a hotel pool and spa [29]. Unfortunately, there is no other information available on this epidemic.

In 2004, an acute gastroenteritis epidemic affected 53 people who had swum in a pool in Vermont, USA. Vomiting and/or diarrhea occurred within 72 h of attending a private indoor pool. Specimens tested positive for NoV. At the time of the inspection, no equipment failures or irregularities were identified. Nevertheless, deficiencies in pool operation and maintenance, including poorly trained operators, inadequate maintenance checks, failure to alert management, and insufficient record keeping were reported [35].

Finally, Yoder and coworkers documented a NoV outbreak linked to a hotel pool in Wisconsin, USA in 2006, with 18 persons exhibiting symptoms of gastroenteritis, related to inadequate disinfection and continued use by ill swimmers [36].

## 4. Occurrence of Enteric Viruses in Swimming Pools under Non-Epidemic Conditions

To date, a limited number of studies examined the extent of viral contamination in swimming pools under non-epidemic conditions.

The first isolation of viruses from urban wading pools was documented in Albany, NY, USA in 1959 [37]. Two enteroviruses (echovirus 3 and echovirus 11) were identified from two chlorinated pools, both filled with water from the municipal supply. The same echovirus strains were found to be present in raw sewage sampled at the Albany treatment plant, reflecting widespread infection in the community.

In Toronto, Canada, Coxsackievirus B1 was isolated from children with pleurodynia, myalgia, and primary peritonitis during 1964. Examination for the virus content of a gauze swab, which was placed daily in a wading pool with high bather load located in a congested city area, revealed the presence of the same Coxsackievirus type [38].

In 1979 in Israel, swimming pool samples were found positive for enterovirus: three for Echovirus 7, two for Coxsackievirus B6, and one for Echovirus 6 [39]. Viruses were isolated from water samples with no detectable fecal or total coliform bacteria.

Different enteroviruses were detected in swimming pools and wading pools equipped with gas chlorine and sand/gravel filters in Texas, USA, in 1980 [40]. After virus concentration from water, samples were assayed on cell culture and plaque assays. Enteroviruses were found in 10/14 (71%) of the examined samples. Coxsackieviruses B3 and B4, poliovirus 1, and echovirus 7 were isolated in pool waters. No correlation was found with total coliform bacteria, as six among the positive virus samples were negative for coliforms. In three samples, viruses were detected in the presence of free chlorine exceeding 0.4 ppm and in the absence of coliforms, indicating that viruses can survive low levels of biocides in actively used pools. Cell cultures used in the study were suited for the isolation of enteroviruses, but it is likely that other viruses, not capable of growing on those cell lines, could also have been present in the water.

Three years later, in 1983, enteroviruses were detected in 28.4% of 116 water samples [41] collected from three outdoor swimming pools. A direct correlation was established between viral and microbial contamination, and the low exchange of water in the pools.

In 2004, van Heerden and coworkers detected HAdV in 12 of 64 samples (18.7%) from an indoor swimming pool and in three of 28 samples (10.7%) from an outdoor swimming pool [7]. Quantitative data were also obtained by Real-time PCR. Application of these results in an exponential risk assessment model, assuming a daily ingestion of 30 mL of water during swimming, indicated a daily risk of infection ranging from 1.92 × 10^−3^ to 3.69 × 10^−3^. No acceptable microbial risk has thus far been established for swimming pool water. However, pool water quality is generally considered comparable to drinking water quality (absence of fecal indicators and pathogens). For this reason, a maximum of one infection per 10,000 consumers per year has been recommended as an acceptable level of microbial risk for swimming pools. The risk of HAdV infections calculated for the swimming pool water in the study exceeded this acceptable risk.

More recently, in 2007 in Cyprus, HAdVs and enteroviruses were detected in public swimming pools complying with bacteriological standards (such as fecal coliforms and enterococci) [42]. The investigation was performed over a period of 21 months, from April 2007 to December 2008. A total of 126 samples were obtained from swimming pools located in five major cities. Bacteriological marker analysis showed that 98% of pools complied with the national regulations. Enteroviruses were identified in four swimming pools, one containing echovirus 18, two containing echovirus 30 and one containing poliovirus Sabin 1. In four swimming pools, HAdVs were detected, all characterized as type 41.

In 2013–2014, a study investigated the presence of human enteric viruses (adenovirus, norovirus, and enterovirus) in indoor and outdoor swimming pool waters in Rome. Bacteriological parameters (fecal indicator bacteria, heterotrophic plate count, *Pseudomonas aeruginosa*, and *Staphylococcus aureus*) were also investigated [43]. Moreover, the study was the first to examine the occurrence of non-enteric viruses in swimming pool waters: human papillomavirus (HPV) and human polyomavirus (HPyV). Interestingly, enteric viruses were not detected, while both HPVs and HPyVs were identified in 9/14 swimming pool water samples, by means of molecular methods. Neither of these viruses had previously been recognized as potential recreational waterborne pathogens, although the WHO Guidelines for safe recreational water environments do include HPVs among non-fecally-derived viruses as viruses associated with plantar warts [3]. A variety of HPVs and HPyVs were found in another study investigating spa/pool waters in Rome [44].

Recently, disinfected water from sixteen pools and spa collected in Rome between 2015 and 2018 were examined for the presence of human enteric viruses (adenovirus, norovirus and enterovirus. Viruses were detected in 25% of the analyzed samples by molecular methods: two samples were positive for adenovirus (type 41) and three samples for norovirus GII (type GII.4) (Bonadonna et al., unpublished data).

## 5. Concluding Remarks

Starting with the first HAdV outbreak recorded in 1951, we reviewed all of the reports concerning swimming-pool related viral illness. The data collected here confirm the involvement of viruses in cases and outbreaks associated with swimming pool attendance.

A number of considerations emerge:

- The paper reviews 29 viral outbreaks due to adenovirus, enterovirus, hepatitis A virus, and norovirus, accounting for more than 3000 cases. Nevertheless, there are likely to have been many other undetected cases and outbreaks. In fact, waterborne diseases are difficult to record because of their wide variety, the difficulty associating symptoms with water use/contact, and the limitations of pathogen detection methods. In the studies described, viruses responsible for reported cases were detected in pool waters only in 21% of the outbreaks, and were found to match with viruses of clinical origin. Currently, better and more rapid methods for the detection of viruses in water samples are available than in the past, resulting in better studies and improved reporting of viral recreational outbreaks worldwide. This allows researchers to identify the causes of outbreaks and possible contributing factors for them. An excellent model to follow is the US-Waterborne Disease and Outbreak Surveillance System (WBDOSS) that has been collecting and reporting data related to occurrences and causes of waterborne disease outbreaks associated with drinking and recreational waters since 1971.
- Some of the studies found that waters meeting state or local water quality requirements contained enteric viruses and were the source of disease outbreaks, confirming that bacterial indicators are unreliable indicators of the presence of viruses and that enteric viruses are important hazardous waterborne pathogens. Indeed, despite the relatively low concentration of viruses in water, they may nevertheless pose health risks due to their low infectious doses (10–100 virions).
- The human illnesses associated with enteric viruses in the reviewed studies were diverse: the most commonly reported symptoms were gastroenteritis, respiratory symptoms, and conjunctivitis. More severe symptoms were also documented, however, including hepatitis and central nervous system infections (aseptic meningitis).
- The majority of the outbreaks described involved mainly children and young people less than 18 years of age. This may be attributable to differences in behaviors, susceptibility and/or immune defenses between children and adults. Children are known to experience more severe symptoms than adults.
- Low concentrations of disinfectant/disinfection malfunction in swimming pools were reported in the vast majority of the outbreaks. Only in one case the concentration of biocide was considered high.

In light of the health hazards posed by swimming pools, it is essential to constantly monitor water quality in swimming pools and to assess the effectiveness of treatment and disinfection processes and compliance with standards. Specifically, appropriate chemical and microbial evaluation of water quality should be carried out, especially when large numbers of bathers are expected to use the pools. Overcrowding should in any case be prevented. Since the behavior of swimmers may affect water quality, strict rules of behavior in the pool should be followed and enforced, including shower before entering the water, wash hands after using the toilet, take children to bathroom before swimming, and, importantly, avoid swimming while sick.

## Figures and Tables

**Table 1:** List of viral swimming pool-related outbreaks.

Caption	N° People Affected	Etiological Agent	Location	Virus Identified in Pool Waters	Year	Reference
Adenovirus	206	HAdV	Colorado, USA	Not tested	1951	[9]
	>300	HAdV 3	Washington	Not detected	1954	[10]
	112	HAdV 3	Canada	Not tested	1955	[11]
	358	HadV 3 and 7	Japan	Not tested	1959	[12]
	45	HAdV 3	Washington	No. Water analyses two weeks after the exposure	1966	[13]
	44	HAdV 7	Kansas	No. Samples were taken after hyperchlorination of the pool	1973	[14]
	105	HAdV 3	Georgia	Not tested	1977	[15]
	72	HAdV 4	Georgia	Yes. First swimming-pool related outbreak in which AdV was recovered from water samples	1977	[16]
	77	HAdV 7a	Oklahoma	Not tested	1982	[17]
	80	Unknow	Greece	Yes. Pool water samples tested found positive for AdVs, and negative for enteroviruses and hepatitis A virus	1995	[18]
	34	HAdV 3	Australia	No	2000	[19]
	59	HAdV 4	Spain	Not tested	2008	[20]
	134	HAdV 3	China	Not tested	2011	[21]
	373	HAdV 7	Taiwan	Not tested	2011	[22]
	55	HAdV 4	China	Yes. Gene sequences obtained from the water samples were 100% identical to the sequences obtained from the swab samples.	2013	[23]
Enterovirus	26	Enterovirus-like	Colorado	Not tested	1987	[24]
	46	Echovirus 30	Ireland	Not tested	1979	[25]
	68	Echovirus 30	Italy	No. Virological analysis of pool waters was performed one month after the outbreak	1997	[26]
	90	Echovirus 3	South Africa	Not tested. Unclean swimming-pool water was confirmed by total coliform count	2001	[27]
	215	Echovirus 13 and 30	Germany	Yes. An echovirus 30 sequence obtained from pond water showed 100% amino-acid homology with sequence obtained from patient isolates	2001	[28]
	36	Echovirus 9	Connecticut	Not tested	2003	[29]
Hepatitis A virus	56	-	Hungary	Not tested	1987	[30]
	20	-	Louisiana, USA	Not tested	1989	[31]
	7	-	Australia	Not tested	1997	[32]
Norovirus	103	-	Ohio	No. Pool water were found negative for both bacterial and viral pathogens.	1977	[33]
	242	-	Finland	Yes. Identical sequence was detected in both patient stool and pool water	2001	[34]
	36	-	Minnesota	Not known	2001–2002	[29]
	53	-	Vermont	Not tested	2004	[35]
	18	-	Wisconsin	Not known	2006	[36]

## References

[1] Sinclair R.G., Jones E.L., Gerba C.P. (2009). Viruses in recreational water-borne disease outbreaks: A review. J. Appl. Microbiol..

[2] Barna Z., Kadar M. (2012). The risk of contracting infectious diseases in public swimming pools. A review. Ann. Ist. Super. Sanità.

[3] WHO: Guidelines for Safe Recreational Water Environments—Swimming Pools and Similar Environments. http://wwwwho.int/iris/handle/10665/43336.

[4] Human Adenovirus Working Group. http://hadvwg.gmu.edu/.

[5] La Rosa G., Suffredini E., Liu D. (2018). Adenovirus. Handbook of Foodborne Diseases.

[6] Mena K.D., Gerba C.P. (2009). Waterborne adenovirus. Rev. Environ. Contam Toxicol..

[7] Van Heerden J., Ehlers M.M., Grabow W.O.K. (2005). Detection and risk assessment of adenoviruses in swimming pool water. J. Appl. Microbiol..

[8] Staggemeier R., Arantes T., Caumo K.S., Rott M.B., Spilki F.R. (2016). Detection and quantification of human adenovirus genomes in Acanthamoeba isolated from swimming pools. An. Acad. Bras. Cienc..

[9] Cockburn T.A. (1953). An epidemic of conjunctivitis in Colorado associated with pharyngitis, muscle pain, and pyrexia. Am. J. Ophthalmol..

[10] Bell J.A., Rowe W.P., Engler J.I., Parrot R.H., Huebner R.J. (1955). Pharyngoconjunctival fever; epidemiological studies of a recently recognized disease entity. J. Am. Med. Assoc..

[11] Ormsby H.L., Aitchison W.S. (1955). The role of the swimming pool in the transmission of pharyngeal-conjunctival fever. Can. Med. Assoc. J..

[12] Fukumi H., Nishikawa M., Takemura M., Odaka Y. (1961). isolation of adenovirus possessing both the antigens of types 3 and 7. Jpn. J. Med. Sci. Biol..

[13] Foy H.M., Cooney M.K., Hatlen J.B. (1968). Adenovirus type 3 epidemic associated with intermittent chlorination of a swimming pool. Arch. Environ. Health.

[14] Caldwell G.G., Lindsey N.J., Wulff H., Donnelly D.D., Bohl F.N. (1974). Epidemic of adenovirus type 7 acute conjunctivitis in swimmers. Am. J. Epidemiol..

[15] Martone W.J., Hierholzer J.C., Keenlyside R.A., Fraser D.W., D’Angelo L.J., Winkler W.G. (1980). An outbreak of adenovirus type 3 disease at a private recreation center swimming pool. Am. J. Epidemiol..

[16] D’Angelo L.J., Hierholzer J.C., Keenlyside R.A., Anderson L.J., Martone W.J. (1979). Pharyngoconjunctival fever caused by adenovirus type 4: Report of a swimming pool-related outbreak with recovery of virus from pool water. J. Infect. Dis..

[17] Turner M., Istre G.R., Beauchamp H., Baum M., Arnold S. (1987). Community outbreak of adenovirus type 7a infections associated with a swimming pool. South. Med. J..

[18] Papapetropoulou M., Vantarakis A.C. (1998). Detection of adenovirus outbreak at a municipal swimming pool by nested PCR amplification. J. Infect..

[19] Harley D., Harrower B., Lyon M., Dick A. (2001). A primary school outbreak of pharyngoconjunctival fever caused by adenovirus type 3. Commun. Dis. Intell..

[20] Artieda J., Pineiro L., Gonzalez M., Munoz M., Basterrechea M., Iturzaeta A., Cilla G. (2009). A swimming pool-related outbreak of pharyngoconjunctival fever in children due to adenovirus type 4, Gipuzkoa, Spain, 2008. Eurosurveillance.

[21] Xie L., Yu X.F., Sun Z., Yang X.H., Huang R.J., Wang J., Yu A., Zheng L., Yu M.C., Hu X.W. (2012). Two adenovirus serotype 3 outbreaks associated with febrile respiratory disease and pharyngoconjunctival fever in children under 15 years of age in Hangzhou, China, during 2011. J. Clin. Microbiol..

[22] Wei S.H. (2016). An Adenovirus Outbreak Associated with a Swimming Facility. SM Trop. Med. J..

[23] Li J., Lu X., Sun Y., Lin C., Li F., Yang Y., Liang Z., Jia L., Chen L., Jiang B. (2018). A swimming pool-associated outbreak of pharyngoconjunctival fever caused by human adenovirus type 4 in Beijing, China. Int. J. Infect. Dis..

[24] Lenaway D.D., Brockmann R., Dola G.J., Cruz-Uribe F. (1989). An outbreak of an enterovirus-like illness at a community wading pool: Implications for public health inspection programs. Am. J. Public Health.

[25] Kee F., McElroy G., Stewart D., Coyle P., Watson J. (1994). A community outbreak of echovirus infection associated with an outdoor swimming pool. J. Public Health Med..

[26] Faustini A., Fano V., Muscillo M., Zaniratti S., La Rosa G., Tribuzi L., Perucci C.A. (2006). An outbreak of aseptic meningitis due to Echovirus 30 associated with attending school and swimming in pools. Int. J. Infect. Dis..

[27] Yeats J., Smuts H., Serfontein C.J., Kannemeyer J. (2005). Investigation into a school enterovirus outbreak using PCR detection and serotype identification based on the 5’ non-coding region. Epidemiol. Infect..

[28] Hauri A.M., Schimmelpfennig M., Walter-Domes M., Letz A., Diedrich S., Lopez-Pila J., Schreier E. (2005). An outbreak of viral meningitis associated with a public swimming pond. Epidemiol. Infect..

[29] Yoder J.S., Blackburn B.G., Craun G.F., Hill V., Levy D.A., Chen N., Lee S.H., Calderon R.L., Beach M.J. (2004). Surveillance for waterborne-disease outbreaks associated with recreational water—United States, 2001–2002. MMWR Surveill. Summ..

[30] Solt K., Nagy T., Csohan A., Csanady M., Hollos I. (1994). An outbreak of hepatitis A due to a thermal spa. Bp. Kozeu..

[31] Mahoney F.J., Farley T.A., Kelso K.Y., Wilson S.A., Horan J.M., McFarland L.M. (1992). An outbreak of hepatitis A associated with swimming in a public pool. J. Infect. Dis..

[32] Tallis G., Gregory J. (1997). An outbreak of hepatitis A associated with a spa pool. Commun. Dis. Intell..

[33] Kappus K.D., Marks J.S., Holman R.C., Bryant J.K., Baker C., Gary G.W., Greenberg H.B. (1982). An outbreak of Norwalk gastroenteritis associated with swimming in a pool and secondary person-to-person transmission. Am. J. Epidemiol..

[34] Maunula L., Kalso S., von Bonsdorff C.H., Ponka A. (2004). Wading pool water contaminated with both noroviruses and astroviruses as the source of a gastroenteritis outbreak. Epidemiol. Infect..

[35] Podewils L.J., Zanardi B.L., Hagenbuch M., Itani D., Burns A., Otto C., Blanton L., Adams S., Monroe S.S., Beach M.J. (2007). Outbreak of norovirus illness associated with a swimming pool. Epidemiol. Infect..

[36] Yoder J.S., Hlavsa M.C., Craun G.F., Hill V., Roberts V., Yu P.A., Hicks L.A., Alexander N.T., Calderon R.L., Roy S.L. (2008). Surveillance for waterborne disease and outbreaks associated with recreational water use and other aquatic facility-associated health events—United States, 2005–2006. MMWR Surveill. Summ..

[37] Kelly S., Sanderson W.W. (1961). Enteric Viruses in Wading Pools. Public Health Rep..

[38] McLean D.M., Larke R.P.B., McNaughton G.A., Best J.M., Smith P. (1965). Enteroviral Syndromes in Toronto, 1964. Can. Med. Assoc. J..

[39] Marzouk Y.M., Goyal S.M., Gerba C. (1980). Relationship of viruses and indicator bacteria in water and wastewater of Israel. Water Res..

[40] Keswick B.H., Gerba C.P., Goyal S.M. (1981). Occurrence of enteroviruses in community swimming pools. Am. J. Public Health.

[41] Cotor F., Zavate O., Finichiu M., Avram G., Ivan A. (1983). Enterovirus contamination of swimming pool water; correlation with bacteriological indicators. Virologie.

[42] Bashiardes S., Koptides D., Pavlidou S., Richter J., Stavrou N., Kourtis C., Papageorgiou G.T., Christodoulou C.G. (2011). Analysis of enterovirus and adenovirus presence in swimming pools in Cyprus from 2007–2008. Water Sci. Technol..

[43] La Rosa G., Della Libera S., Petricca S., Iaconelli M., Briancesco R., Paradiso R., Semproni M., Di Bonito P., Bonadonna L. (2015). First detection of papillomaviruses and polyomaviruses in swimming pool waters: Unrecognized recreational water-related pathogens?. J. Appl. Microbiol..

[44] Di Bonito P., Iaconelli M., Gheit T., Tommasino M., Della Libera S., Bonadonna L., La Rosa G. (2017). Detection of oncogenic viruses in water environments by a Luminex-based multiplex platform for high throughput screening of infectious agents. Water Res..

